# 硼替佐米、利妥昔单抗、环磷酰胺、阿霉素和泼尼松（VR-CAP）方案治疗初治套细胞淋巴瘤60例的疗效及预后分析

**DOI:** 10.3760/cma.j.issn.0253-2727.2021.05.011

**Published:** 2021-05

**Authors:** 怡文 曹, 重 郑, 彭鹏 许, 澍 程, 黎 王, 樱 钱, 维莅 赵

**Affiliations:** 上海血液学研究所，医学基因组学国家重点实验室，国家转化医学中心（上海），上海交通大学医学院附属瑞金医院 200025 Shanghai Institute of Hematology, State Key Laboratory of Medical Genomics, National Research Center for Translational Medicine at Shanghai, Ruijin Hospital Affiliated to Shanghai Jiao Tong University School of Medicine, Shanghai 200025, China

套细胞淋巴瘤（MCL）占所有非霍奇金淋巴瘤（NHL）的2.5％～6.0％，经典型MCL临床病程常呈惰性且伴多部位结外累及[1]–[2]。部分MCL可出现形态学变异，其中以母细胞变异型最常见[3]–[4]，母细胞变异型MCL临床病程常为侵袭性且多伴有骨髓及脾脏累及[5]。关于MCL的分子学发病机制研究认为，染色体t（11;14）（q13;q32）易位产生过表达的CyclinD1导致细胞周期紊乱及肿瘤的发生[6]。除细胞周期紊乱外，DNA损伤导致ATM、CHK2、TP53等基因突变及相关细胞生存途径的激活（如mTOR、NF-κB及NOTCH等）在MCL发病机制中均发挥重要作用，其中TP53基因突变是影响MCL患者疗效及预后的危险因素[7]–[8]。目前R-CHOP（利妥昔单抗、环磷酰胺、阿霉素、长春新碱和泼尼松）为基础的免疫治疗联合化疗方案是不适宜强化疗及无移植指征MCL患者的基础治疗方案[9]–[10]，但在亚洲人群中进行的回顾性研究显示患者在该方案中的疗效获益及长期生存获益均有限[11]–[15]。蛋白酶体抑制剂硼替佐米已被多国批准用于MCL的一线/复发治疗[16]–[18]。一项关于VR-CAP方案（硼替佐米、利妥昔单抗、环磷酰胺、阿霉素和泼尼松）治疗MCL的Ⅲ期临床研究（LYM-3002）显示，VR-CAP方案治疗MCL的完全缓解率、4年无进展生存率及总生存期均优于R-CHOP方案，且治疗兼具可控性和安全性[19]。为了进一步评估VRCAP方案治疗中国MCL患者的疗效及预后，我们将2015年4月至2020年3月收治的60例以VR-CAP方案治疗的初治MCL患者临床资料及生存情况进行总结分析，探讨VR-CAP方案在中国MCL患者治疗中的疗效及预后情况。

## 病例与方法

1. 病例资料：回顾性分析2015年4月至2020年3月本院收治的初治MCL患者60例，均经病理活检诊断为MCL，符合WHO 2008年淋巴瘤分类标准[20]。所有患者均行PET-CT及骨髓穿刺活检，并根据MCL国际预后评分系统联合Ki-67指数（MIPI-c）进行预后分层[21]–[22]。

2. 治疗方案：初治MCL患者均接受标准VR-CAP方案6个疗程（图1），该方案包括：硼替佐米1.3 mg·m^−2^·d^−1^，静脉注射，第1、4、8、11天；利妥昔单抗375 mg/m^2^，静脉滴注，第0天；环磷酰胺750 mg/m^2^，静脉滴注，第1天；阿霉素50 mg/m^2^，静脉注射，第1天；泼尼松100 mg·m^−2^·d^−1^，口服，第1～5天。维持治疗阶段为利妥昔单抗375 mg/m^2^，静脉滴注，每3个月1次。

**图1:**
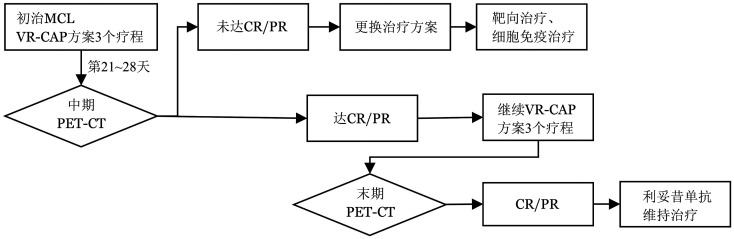
VR-CAP方案治疗初治套细胞淋巴瘤（MCL）流程 VR-CAP方案：硼替佐米、利妥昔单抗、环磷酰胺、阿霉素、泼尼松；CR：完全缓解；PR：部分缓解

3. 疗效评估：患者在完成3次VR-CAP方案后进行中期疗效评估，6次治疗后进行末期疗效评估，所有患者均行PET-CT并根据2014版Lugano疗效评估标准[23]判定疗效，疗效评估分为完全缓解（CR）、部分缓解（PR）、疾病稳定（SD）、进展和复发（PD），有效为CR+PR。

4. 统计学处理：采用SPSS 22.0进行统计学分析，各分类变量组间比较采用*χ*^2^检验或Fisher精确概率法，采用Kaplan-Meier法描绘生存曲线，采用Log-rank检验比较不同生存曲线组间差异，采用Cox回归模型进行单因素分析，将单因素分析中差异有统计学意义的因素纳入多因素分析。以双侧*P*<0.05表示差异有统计学意义。

## 结果

1. 一般资料：详见表1。60例患者中，男47例（78.3％）、女13例（21.7％），男女比为3.6∶1。中位发病年龄60（36～75）岁，其中年龄≥60岁者31例（51.7％）。Ann Arbor分期Ⅲ～Ⅳ期者52例（86.7％）。根据美国东部肿瘤协作组（ECOG）评分≥2分者5例（8.3％）。初治时有骨髓累及者22例（36.7％）；血清LDH水平升高（>192 U/L）者24例（40.0％）；WBC升高（≥10×10^9^/L）者9例（15.0％）；Ki-67≥30％者39例（65.0％）；根据MIPI-c进行分组，低危组/低中危38例（63.3％），高中危组/高危组22例（36.7％）。

**表1:** VR-CAP方案治疗套细胞淋巴瘤有无获得完全缓解（CR）患者基线资料比较［例（％）］

临床特征	例数	疗效分组		*P*值
		达CR组（46例）	未达CR组（14例）	
性别				0.444
男	47	35（74.5）	12（25.5）	
女	13	11（84.6）	2（15.4）	
年龄				0.021
<60岁	29	26（89.7）	3（10.3）	
≥60岁	31	20（64.5）	11（35.5）	
AnnArbor分期				0.094
Ⅰ～Ⅱ	8	8（100.0）	0（0.0）	
Ⅲ～Ⅳ	52	38（73.1）	14（26.9）	
骨髓累及				0.473
无	38	28（73.7）	10（26.3）	
有	22	18（81.8）	4（18.2）	
ECOG评分				0.141
0～1分	55	44（80.0）	11（20.0）	
2～5分	5	2（40.0）	3（60.0）	
LDH				0.383
≤192 U/L	36	29（80.6）	7（19.4）	
>192 U/L	24	17（70.8）	7（29.2）	
WBC				0.423
<10×10^9^/L	51	40（78.4）	11（21.6）	
≥10×10^9^/L	9	6（66.7）	3（33.3）	
Ki-67				0.002
<30％	21	21（100.0）	0（0.0）	
≥30％	39	25（64.1）	14（35.9）	
MIPI-c				0.025
低危/低中危	38	33（86.8）	5（13.2）	
高中危/高危	22	13（59.1）	9（40.9）	

注：VR-CAP方案：硼替佐米、利妥昔单抗、环磷酰胺、阿霉素、泼尼松；ECOG评分：美国东部协作组体能状态评分；MIPI-c：套细胞淋巴瘤国际预后评分系统联合Ki-67指数

2. 疗效：VR-CAP方案治疗后，初治达CR者46例（76.7％），初治达PR者8例（13.3％），总有效率（ORR）为90.0％。其中年龄≥60岁（*P*＝0.021）、Ki-67≥30％（*P*＝0.002）及MIPI-c分层高中危/高危组（*P*＝0.025）是影响患者疗效的因素（表1）。经3个疗程治疗后中期评估未达CR或PR者6例（10.0％），均为男性，Ann Arbor分期均为Ⅲ～Ⅳ期且Ki-67均≥30％，其中5例年龄≥60岁。中位随访时间21.3（3～63）个月，2年无进展生存（PFS）率为（73.2±3.3）％，2年总生存（OS）率为（82.3±3.1）％（图2）。

**图2:**
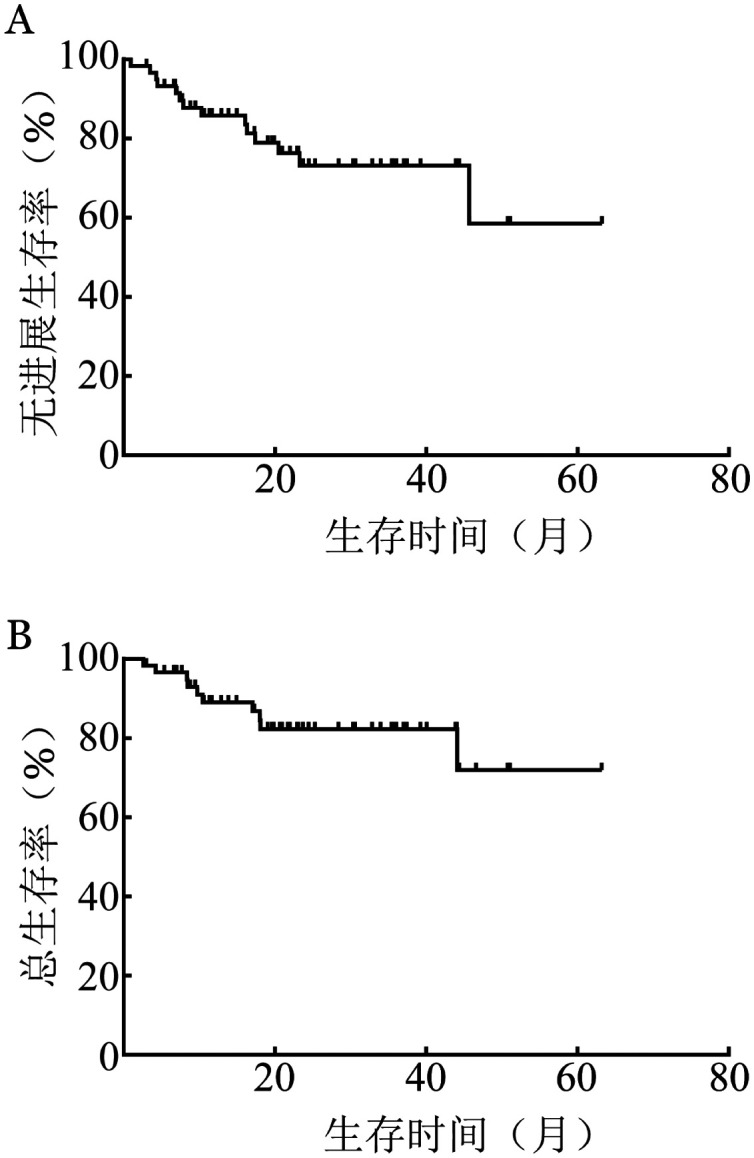
VR-CAP方案治疗60例套细胞淋巴瘤患者无进展生存（A）及总生存（B）曲线 VR-CAP方案：硼替佐米、利妥昔单抗、环磷酰胺、阿霉素、泼尼松

3. 影响患者生存的预后因素分析：单因素分析中，ECOG评分≥2分（*P*＝0.029，*P*＝0.004），外周血WBC升高（*P*＝0.041，*P*＝0.004），及MIPI-c分层高中危/高危组（*P*＝0.010，*P*＝0.007）影响患者PFS及OS；Ki-67≥30％（*P*＝0.022）影响患者PFS，年龄≥60岁（*P*＝0.049）影响患者OS。将单因素*P*<0.05的因素纳入多因素分析，MIPI-c高中危/高危为影响患者PFS（*HR*＝4.050，95％*CI* 1.316～12.465，*P*＝0.015）及OS（*HR*＝8.543，95％*CI* 1.768～41.275，*P*＝0.008）的独立危险因素（表2、表3）。

**表2:** VR-CAP方案治疗套细胞淋巴瘤患者预后的单因素分析（*x*±*SE*）

因素	例数	无进展生存			总生存		
		率	*χ*^2^值	*P*值	率	*χ*^2^值	*P*值
性别			1.068	0.301		0.919	0.338
男性	47	43.8±3.6			50.6±3.7		
女性	13	45.6±3.7			47.2±3.6		
年龄			2.917	0.088		3.861	0.049
<60岁	29	44.5±3.0			47.7±2.2		
≥60岁	31	38.7±4.9			46.3±4.9		
AnnArbor分期			0.040	0.950		0.281	0.596
Ⅰ～Ⅱ	8	35.6±5.1			35.2±5.3		
Ⅲ～Ⅳ	52	44.2±3.5			52.9±3.2		
骨髓累及			0.660	0.416		1.798	0.18
无	38	46.8±3.7			54.7±3.4		
有	22	37.3±4.6			39.6±4.4		
ECOG评分			4.792	0.029		8.427	0.004
0～1	55	45.3±3.3			53.8±3.0		
2～5	5	9.4±2.6			9.8±2.3		
LDH			0.136	0.712		1.087	0.297
≤192 U/L	36	44.9±4.1			54.4±3.5		
>192 U/L	24	39.3±4.1			41.4±3.8		
WBC			4.190	0.041		8.310	0.004
<10×10^9^/L	51	46.0±3.6			55.3±3.0		
≥10×10^9^/L	9	26.8±8.3			27.6±7.7		
Ki-67			5.218	0.022		2.567	0.109
<30％	21	55.1±4.0			59.8±3.1		
≥30％	39	35.8±3.4			41.0±2.9		
MIPI-c			6.620	0.010		7.220	0.007
低危/低中危	38	48.8±3.7			58.0±2.7		
高中危/高危	22	30.0±5.4			33.5±5.1		

注：VR-CAP方案：硼替佐米、利妥昔单抗、环磷酰胺、阿霉素、泼尼松；ECOG评分：美国东部肿瘤协作组体能状态评分；MIPI-c：套细胞淋巴瘤国际预后评分系统联合Ki-67指数

**表3:** VR-CAP方案治疗套细胞淋巴瘤患者多因素预后分析

影响因素	无进展生存			总生存		
	*HR*	95％*CI*	*P*值	*HR*	95％*CI*	*P*值
年龄（<60岁，≥60岁）				2.117	0.309～14.525	0.445
ECOG评分（0～2分，3～5分）	3.372	0.657～17.305	0.145	5.459	0.788～37.811	0.086
WBC（<10×10^9^/L，≥10×10^9^/L）	1.606	0.381～6.776	0.519	1.362	0.257～7.218	0.716
Ki-67（<30％，≥30％）	4.927	0.600～40.484	0.138			
MIPI-c（低危/低中危，高中危/高危）	4.050	1.316～12.465	0.015	8.543	1.768～41.275	0.008

注：VR-CAP方案：硼替佐米、利妥昔单抗、环磷酰胺、阿霉素、泼尼松；ECOG评分：美国东部协作组体能状态评分；MIPI-c：套细胞淋巴瘤国际预后评分系统联合Ki-67指数

4. 基因突变对预后的影响：60例患者中39例（65.0％）完成肿瘤组织基因检测，8例（20.5％）检出TP53基因突变，CCND1基因突变7例（17.9％），ATM基因突变6例（15.4％），NOTCH1、TNFAIP3及CARD11基因突变各3例（7.7％）。其中TP53基因突变患者初治后仅3例达CR（37.5％），中位PFS期仅8（95％*CI* 4.628～13.872）个月，中位OS期仅10（95％*CI* 6.862～16.488）个月，与未检测到TP53基因突变患者的PFS及OS期（均未达到）差异均有统计学意义（*P*值均<0.001）（图3）。其他基因突变与患者疗效及预后差异均无统计学意义。

**图3:**
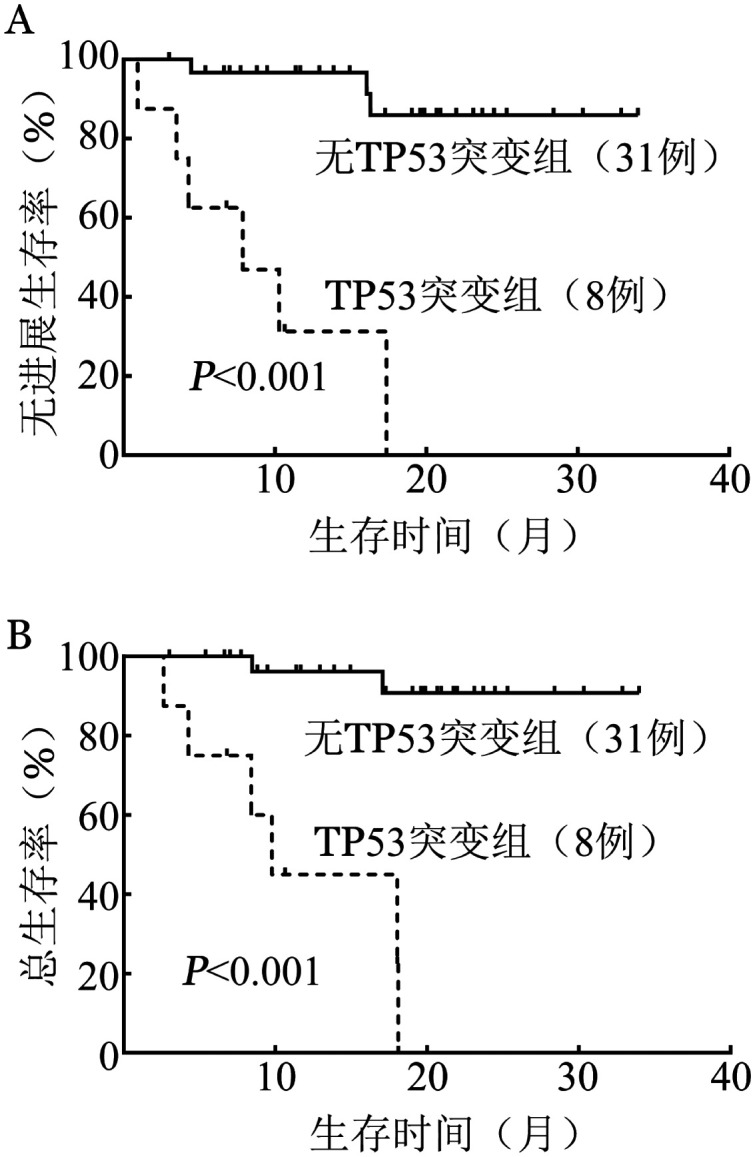
VR-CAP方案治疗TP53突变与无TP53突变套细胞淋巴瘤患者无进展生存（A）及总生存（B）曲线 VR-CAP方案：硼替佐米、利妥昔单抗、环磷酰胺、阿霉素、泼尼松

## 讨论

本研究是一项在中国人群中进行的VR-CAP方案治疗MCL的临床研究分析，首次评估了中国初治MCL患者使用硼替佐米、利妥昔单抗联合化疗的疗效及预后。硼替佐米是一种蛋白酶体抑制剂，欧盟、美国及其他几个国家/地区已批准其用于MCL的一线及二线治疗[16]–[17]，但目前国内关于硼替佐米治疗初治MCL的临床研究报道较少。本研究中VR-CAP方案治疗初治MCL患者的中位发病年龄60岁，男性占78.3％，86.7％的患者发病时Ann Arbor分期为Ⅲ～Ⅳ期，与近年国际临床试验报道相似[5],[19]。本研究中VR-CAP方案治疗MCL的ORR达90.0％，CR率达76.7％。分组分析显示，患者疗效不佳与初治时年龄≥60岁，Ki-67≥30％及MIPI-c分层高中危/高危组有关。而LDH升高及骨髓受累均未影响患者疗效，这与早前国内外使用R-CHOP、CHOP或其他无硼替佐米的治疗方案结论不同[24]–[25]，后续可进行多中心及前瞻性临床研究进一步讨论VR-CAP方案对LDH升高及有骨髓累及MCL患者疗效的影响。

早前的国际多中心LYM-3002临床研究[5]报道了VR-CAP方案治疗初治MCL的中位OS期显著高于R-CHOP方案，其中VR-CAP组2年OS率约为83％，本研究结果与之相似。本研究中预后因素分析显示，VR-CAP方案治疗后Ki-67≥30％与Ki-67<30％的患者OS差异无统计学意义，在LYM-3002临床研究中同样报道了相关结论，提示Ki-67阳性的MCL患者可能从VR-CAP方案中获益，后续仍需进行多中心临床研究进一步论证上述结论。LYM-3002临床研究进一步对中东亚地区MCL患者进行亚组分析，接受VR-CAP方案的东亚患者与接受R-CHOP方案相比PFS率改善了43％（*HR*＝0.7，*P*＝0.157）。东亚地区人数较少可能是导致差异无统计学意义的原因，但使用VR-CAP方案的MCL患者PFS期仍有明显提高[19]。因此VR-CAP方案对于初治的中国MCL患者是一种积极的治疗选择。进一步对本研究中患者预后分层分析，MIPI-c高中危/高危组PFS及OS均与低危/低中危组有显著差异，是影响患者预后的独立危险因素，这与LYM-3002临床研究[5]的结论相似，提示对于MIPI-c分层高中危/高危组患者，未来仍需寻找更加个体化的治疗方案。

本研究60例MCL患者中39例完成基因检测，常见突变基因依次为TP53（8例，20.5％）、CCND1（7例，17.9％）、ATM（6例，15.4％）。其中TP53基因突变患者使用VR-CAP方案未能获得理想的疗效及长生存。8例（20.5％）存在TP53突变患者中位PFS期仅8个月，中位OS期仅10个月，是VRCAP方案治疗MCL患者的预后不良因素。多个国际临床研究与报道认为TP53基因突变与患者预后相关[26]–[28]，具有17p缺失或TP53突变的MCL患者OS期明显短于无17p缺失或TP53突变患者[26],[29]。近年在北欧年轻患者中进行的MCL2和MCL3临床试验[30]中TP53突变的年轻患者中位OS期仅1.8年。目前关于TP53突变的MCL患者无标准一线治疗方案，近年多项MCL相关临床研究均不能改善TP53突变患者的不良预后[30]–[32]，仅少数研究认为BTK抑制剂联合BCL-2抑制剂能改善TP53突变MCL患者的疗效[33]。后续我中心将进一步探索靶向药物在有预后不良分子学标志物MCL患者治疗中的应用时机。本研究中突变频率第二位基因为ATM，ATM是MCL的主要致病基因，通过抑癌基因编码DNA损伤信号相关蛋白质。早前研究认为ATM突变对于MCL患者的整体生存无影响[34]，本研究结果与之相符。

总之，MCL是具有高度侵袭性及异质性的一类NHL，在目前免疫治疗联合化疗的时代，VR-CAP方案能显著提高患者的疗效及生存，但对于预后分层高危患者及有特定基因突变患者仍需寻找更加个体化治疗方案，并进一步探索分子靶向药物的使用时机，以进一步提高MCL患者的整体疗效及长期生存。
